# What do ALS motor phenotypes measure? A population-based decomposition of their prognostic dimensions

**DOI:** 10.1007/s00415-026-14134-z

**Published:** 2026-09-18

**Authors:** Andrea Calvo, Cristina Moglia, Stefano Callegaro, Antonio Canosa, Umberto Manera, Gabriele Mora, Sara Cabras, Francesca Palumbo, Enrico Matteoni, Alessandra Maccabeo, Giorgio Pellegrino, Emilio Minerva, Daniela Pascariu, Fabrizio D’Ovidio, Rosario Vasta, Letizia Mazzini, Fabiola De Marchi, Adriano Chiò

**Affiliations:** 1 ‘Rita Levi Montalcini’ Department of Neuroscience, University of Torino, via Cherasco 15, 10126 Turin, Italy; 2 Azienda Ospedaliero Universitaria Città Della Salute E Della Scienza, Turin, Italy; 3 Department of Translational Medicine, University of Eastern Piedmont, Novara, Italy; 4 Azienda Ospedaliero Universitaria Maggiore Della Carità Novara, Novara, Italy

**Keywords:** Amyotrophic lateral sclerosis, Motor phenotype, Prognosis, Population-based registry, Survival analysis, Disease staging

## Abstract

**Background:**

The ALS-OPM 3.3 classification stratifies incident ALS through three axes: onset region (O), propagation time (P) and motor-neuron pattern (M). We assessed whether OPM 3.3 improves prognostic discrimination over established classifications and characterised how it decomposes their prognostic signal in a population-based cohort.

**Methods:**

In the prospective Piedmont and Aosta Valley ALS Register (2000–2022), patients were classified on the three axes; M was operationalised as M_3class (M0 balanced, M1d UMN-predominant, M2d LMN-predominant), with the PLS-spectrum M1p analysed separately. Outcome was survival (death or tracheostomy) from onset. Multivariable Cox models (O, P1(n), M_3class, age, sex) underwent bootstrap optimism-correction and fivefold cross-validation, and were compared against alternative taxonomies and clinical staging systems.

**Results:**

Amongst 2,738 non-PLS patients (2,561 events, 93.5%), median survival differed across M classes (M0 27.0, M2d 36.0, M1d 41.9 months). OPM 3.3 outperformed the bulbar/spinal dichotomy but matched the classical phenotype (optimism-corrected C-index 0.703, 95% CI 0.685–0.719 vs 0.701). In the arm-proximal cell (95.7% M2d), the O2p protective effect vanished after M adjustment (HR 0.99, 95% CI 0.79–1.24), whilst M2d retained HR 0.83 (95% CI 0.72–0.95), showing prognosis reflects motor-neuron pattern, not anatomy. Adding King’s, Milano-Torino (MiToS), or Fine’til 9 (FT9) clinical staging further improved discrimination.

**Conclusions:**

OPM 3.3 did not improve discrimination over established phenotypes but decomposed their prognostic signal into independently interpretable dimensions. Favourable prognosis of arm-proximal/flail-arm onset reflects the underlying LMN-predominant phenotype, not onset site. The motor-neuron axis is robust to the treatment of the propagation axis and independent of clinical staging.

**Supplementary Information:**

The online version contains supplementary material available at https://doi.org/10.1007/s00415-026-14134-z.

## Introduction

Amyotrophic lateral sclerosis (ALS) is a clinically heterogeneous neurodegenerative disorder of upper and lower motor neurons [1–3]. Median survival from symptom onset is approximately 30 months in incident population-based registries, but the dispersion is wide [4, 5]. Multiple demographic and clinical factors influence survival, including age at onset, site of onset, and rate of disease progression [6]; cognitive and behavioural impairment, present in a substantial proportion of patients, is a further recognised prognostic factor, cognitive change predicting functional decline over the disease course [7, 8]. A robust prognostic stratification at first observation is therefore central to clinical counselling, trial design, and clinical-trial enrichment.

Multiple stratification systems have been proposed in the past two decades. The classical bulbar/spinal taxonomy is parsimonious but coarse [9]; the El Escorial/Awaji-Shima criteria classify diagnostic certainty rather than prognosis [10]; more recently, the Gold Coast criteria have simplified diagnosis to a single category, increasing sensitivity by removing the graded levels of diagnostic certainty [11]; the King’s and Milano-Torino (MiToS) staging systems, and the more recent Fine’til 9 (FT9) system, describe the extent of disease reached rather than the phenotype at first observation [12–14]; predictive prognostic indices such as ENCALS [15] combine multiple variables but lack a phenotypic structure. Meyer et al. [16, 17] recently proposed the OPM classification (2025, with a revised ALS-OPM 3.3 specification in 2026), a three-axis system that summarises an individual ALS patient at first observation by Onset region (O), Propagation time (P), and Motor-neuron pattern (M), preserving the descriptive clarity of phenotypic taxonomies whilst embedding a prognostic structure. Whether this tridimensional architecture formalises recognised phenotypes into separable, prognostically non-redundant axes, rather than simply outperforming simpler taxonomies, is the empirical question that the present population-based incident cohort allows us to test. The three OPM dimensions differ in their temporal properties. O is a fixed historical attribute reflecting the anatomical site of symptom onset. M is a cross-sectional descriptor of motor-neuron involvement at clinical assessment. In contrast, P is intrinsically longitudinal, because disease propagation can only be observed after spread has occurred. Therefore, unlike O and M, complete assignment of P categories may require follow-up and should not be interpreted as a purely baseline characteristic.

The objectives of this work are: (i) to characterise the full ALS-OPM 3.3 classification on the PARALS incident cohort 2000–2022 under TRIPOD-compliant internal validation; (ii) to characterise the tridimensional structure of OPM by quantifying the cross-axis information of O × M and the contributions of P, with explicit attention to the cells where the three axes overlap or complement each other; and (iii) to position OPM 3.3 against alternative phenotypic taxonomies (bulbar/spinal, El Escorial, classical phenotypes), and against clinical staging systems (King’s, MiToS, FT9), testing whether OPM phenotype at first observation captures prognostic information that is independent of, and complementary to, disease stage at presentation.

## Methods

### Study design and population

PARALS is a prospective, population-based registry of incident ALS cases in the Piemonte and Aosta Valley regions of north-west Italy [4, 5]. All patients diagnosed between 1 January 2000 and 31 December 2022 were eligible (*N* = 2808 unique patients with non-cognitive onset). OPM descriptors were assigned to every patient from registry data, further verified against original clinical charts.

### OPM classification

Each patient was stratified according to the ALS-OPM 3.3 specification [16, 17] across three axes. Onset (O) was taken from the region of first motor involvement, yielding eight categories: O1 head/bulbar; O2d arm, distal; O2p arm, proximal/scapulohumeral; O2x arm, mixed; O3r trunk/respiratory; O4d leg, distal; O4p leg, proximal; O4x leg, mixed. Propagation (P1(n)) was the interval in months from symptom onset to involvement of a second, vertically distant body region, capped at 60 months for the Cox specification. Following the ALS-OPM 3.3 specification [17], its temporal qualifier was dated primarily from the patient’s history (the anamnestic date of second-region involvement). For patients in whom propagation had not yet occurred at the first visit, it was dated prospectively from the first follow-up ALSFRS-R [18] assessment at which a non-onset region subscore fell below 4. Because the anamnestic date of second-region involvement depends on patient recall, and recall may be less reliable in patients assessed several years after onset, we cross-checked it against the prospective ALSFRS-R-based date wherever both were available; the two sources were concordant in the large majority of cases (Supplementary Table S1), and the landmark analysis (Supplementary Table S6), which does not depend on the exact timing of propagation, confirmed the direction and significance of the propagation effect. Patients with established propagation but no datable timing were classified P1(x). Concordance between the anamnestic and prospective timing sources is reported in Supplementary Table S1. Motor-neuron pattern (M) was operationalised as a three-level variable M_3class (M0, balanced UMN+LMN; M1d, UMN-predominant; M2d, LMN-predominant, comprising the proximal and other non-classic lower motor-neuron presentations, chiefly the scapulohumeral ‘flail-arm’ and ‘flail-leg’ variants), covering 99% of the non-PLS analytic cohort; the PLS-spectrum M1p (*n* = 64) was analysed separately to avoid immortal-time bias. Full category definitions, the derivation of P1(n), and the cross-axis correlation analysis are provided in the Supplementary Methods. O and M are baseline descriptors, whereas P is a longitudinal descriptor incorporated into the OPM framework.

### Statistical methods

The primary outcome was the composite of death or invasive ventilation by tracheostomy, measured in months from symptom onset; throughout, ‘events’ denotes patients reaching this composite endpoint. Censoring date was 31 December 2025. Survival differences between M classes were tested by the multivariate log-rank test.

Multivariable Cox proportional-hazards models were fitted with O2d and M0 as reference categories: O2d, distal arm onset, because it is the most common typical spinal-onset presentation and, therefore, the natural comparator for the other onset regions, and M0, the balanced upper and lower motor-neuron pattern, because it represents the classical Charcot form of ALS against which the upper- and lower-predominant variants are most naturally contrasted. The association between O and M was quantified by Cramér’s V, and their mutual prognostic independence by nested likelihood-ratio tests. The primary specification OPM5 (OPM_O 8-cat + P1(n) linear + M_3class + age + sex) was fitted on the analytic dataset (*N* = 2738, no missingness; M1p excluded); an extended model with a natural cubic spline on P (OPM6) was also evaluated. Discrimination was quantified by Harrell’s C-index, the probability that, of two patients, the one who survives longer had the lower predicted risk, so that 0.5 denotes chance and 1.0 perfect ranking, and internally validated via bootstrap optimism-correction using the Harrell–Steyerberg procedure with 200 resamples for the primary models, and repeated fivefold cross-validation with 20 repetitions, following TRIPOD [19–22]. For the comparison of alternative taxonomies, in which several nested models were re-estimated on each resample, 120 resamples were used, the smaller number reflecting the higher computational cost of the nested re-estimation. Competing models were compared by the Akaike Information Criterion (AIC), which ranks models by fit whilst penalising the number of parameters, a lower value indicating a better fit-parsimony balance. Software, reference framework, and detailed algorithmic steps are reported in the Supplementary Methods.

## Results

### Cohort

Of the 2808 incident PARALS patients with non-cognitive onset, 2802 had complete date-of-onset and outcome ascertainment. The analytic dataset for the primary OPM Cox model—patients with complete OPM_O, P1(n), M_3class, age, sex, and outcome, excluding the *n* = 64 M1p PLS-spectrum subgroup—comprised *N* = 2738 patients (events 2561, 93.5%; median age at onset 67.8 years, IQR 59.8–74.4) (Supplementary Figure S1). The OPM_O distribution is detailed in Table 1.

**Table 1:** Topographic distribution (OPM_O × M_3class) and survival by motor class on the primary analytic dataset (M1p excluded; *N* = 2738; events = 2561; row percentages within OPM_O)

OPM_O	Topographic interpretation	*N*	M0% (n)	M1d % (n)	M2d % (n)
O1	Head/bulbar	906	89.0% (806)	11.0% (100)	—
O2d	Arm — distal classic	608	90.8% (552)	7.9% (48)	1.3% (8)
O2p	Arm — proximal	163	1.8% (3)	2.5% (4)	95.7% (156)
O2x	Arm — mixed/non-classifiable	29	82.8% (24)	10.3% (3)	6.9% (2)
O3r	Trunk/respiratory	62	100% (62)	—	—
O4d	Leg — distal pattern	863	33.3% (287)	20.9% (180)	45.9% (396)
O4p	Leg — proximal pattern	91	62.6% (57)	16.5% (15)	20.9% (19)
O4x	Leg — mixed/non-classifiable	16	37.5% (6)	18.8% (3)	43.8% (7)
	Total	2738	1797 (65.6%)	353 (12.9%)	588 (21.5%)
	Median survival, months		27.0	41.9	36.0
	Event %		94.4	88.1	94.0
	Median age, years (IQR)		68.6 (60.8–75.2)	65.0 (54.9–72.2)	66.8 (59.8–72.9)

Survival, event rate, and age are shown per M class in the lower panel; comparison of survival across the three M classes: multivariate log-rank *χ*^2^ = 121.2 (df = 2), *p* = 4.8 × 10⁻^2^⁷

### M_3class distribution and survival on the full cohort

**Fig. 1:**
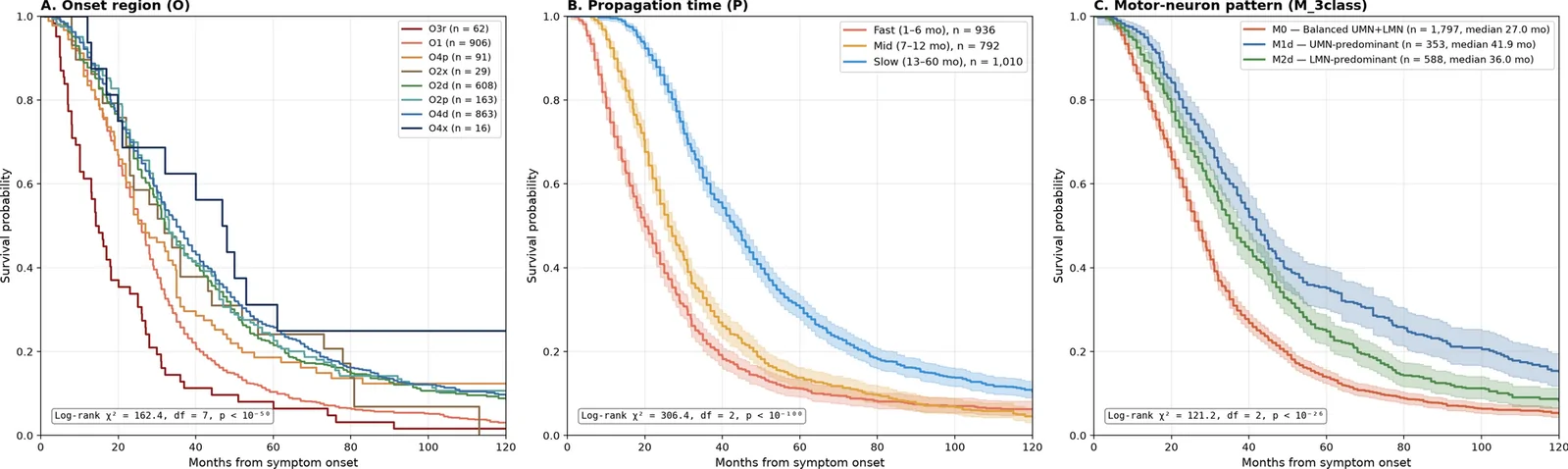
The three axes of ALS-OPM 3.3 on the PARALS analytic cohort (*N* = 2738 non-PLS, events = 2561; M1p excluded). Kaplan–Meier survival curves stratified by each of the three OPM axes. **a** Survival by onset region (O), the eight native ALS-OPM 3.3 categories (O1 head/bulbar, O2d/p/x arm distal/proximal/mixed, O3r trunk-respiratory, O4d/p/x leg distal/proximal/mixed); median survival ranges from 14 months (O3r, respiratory onset, the highest-risk category) to 47 months (O4x, leg-mixed onset); the apparent advantage of O4x should be interpreted with caution given its small size (*n* = 16, the smallest onset category) and the wide, non-significant adjusted hazard ratio (Table 2). **b** Survival by propagation time (P), shown as tertiles of P1(n) capped at 60 months: fast (P1 1–6 months, *n* = 936), mid (P1 7–12 months, *n* = 792), slow (P1 13–60 months, *n* = 1010); medians 21.0, 27.0 and 43.1 months, respectively. Propagation time is the dominant quantitative prognostic axis of OPM 3.3 (the largest between-group survival contrast on this cohort). **c** Survival by motor-neuron pattern (M), operationalised as M_3class on PARALS: M0 balanced UMN+LMN (*n* = 1797, median 27.0 months), M1d dominant UMN (*n* = 353, median 41.9 months), and M2d dominant LMN with scapulohumeral (flail-arm) and flail-leg substrates (*n* = 588, median 36.0 months). Multivariate log-rank for M_3class: χ^2^ = 121.2 (df = 2), *p* < 10⁻^2⁶^. The three panels show that the three axes carry distinct prognostic signals: anatomy of onset (O, panel a), propagation kinetics (P, panel b), and motor-neuron pattern (M, panel c), which combine to define the three-axis ALS-OPM 3.3 architecture established in this work

The three-level M classification was applicable to 100.0% of the non-PLS registry. On the full non-PLS survival cohort (*N* = 2738), the three classes had clearly distinct sizes and survival (Table 1, Fig. 1c): the balanced UMN+LMN bulk M0 represents about two-thirds of the cohort and has the shortest median survival, the proximal/atypical LMN-predominant M2d an intermediate prognosis, and the dominant-UMN M1d the longest. The difference was highly significant (multivariate log-rank *χ*^2^ = 121.2, df = 2, *p* = 4.8 × 10⁻^2^⁷; all three pairwise comparisons *p* < 10⁻^4^), confirming that the three populations are distinct.

### OPM_O × M_3class joint distribution

The cross-tabulation (Table 1) shows that the two axes carry partly correlated but non-redundant information. Three OPM_O categories have near-deterministic M_3class composition (O1 essentially M0 + M1d with no M2d; O3r entirely M0; O2p almost entirely M2d, 95.7%), whereas the most populous categories (O2d arm-distal, O4d leg-distal) and the rarer mixed categories (O2x, O4x) span all three M classes; O4d is the cell where M_3class adds most prognostic resolution beyond OPM_O. The association is moderate (Cramér’s V = 0.51; *χ*^2^ = 1454, *p* < 10⁻^300^), yet each axis retains independent prognostic value: adjusted for age and sex, O contributes beyond M (likelihood-ratio *χ*^2^ = 57.5, df = 7, *p* < 10⁻⁹) and M beyond O (*χ*^2^ = 75.7, df = 2, *p* < 10⁻^16^).

### Cox prognostic model with M_3class

Hierarchical Cox models on the analytic dataset (Supplementary Table S2) show the contribution of each component of the primary OPM specification. The full primary model OPM5 (OPM_O 8-cat + P1(n) linear + M_3class + age + sex) reaches an apparent C-index of 0.7056. An extended model with a natural cubic spline on P1(n) (OPM6), a natural cubic spline being a flexible curve that allows a non-linear relationship between propagation time and risk, further improves apparent C only marginally to 0.7083 (corrected 0.7049; likelihood-ratio test versus OPM5 *χ*^2^ = 5.56, df = 3, *p* = 0.14), indicating that the linear specification of P1(n) capped at 60 months captures most of the prognostic signal.

### Hazard ratios in the refined OPM model (OPM5)

Adjusted hazard ratios are reported in Table 2 and visualised in Supplementary Figure S2. The reference category for OPM_O is O2d (arm distal, the most common typical spinal-onset category); the reference category for M_3class is M0 (classical mixed).

**Table 2:** Hazard ratios in the refined OPM primary model OPM5 (M_3class, M1p excluded; *N* = 2738; events = 2561)

Variable	HR	95% CI	*p* value
OPM_O (ref = O2d, arm distal)			
O1 (head/bulbar)	1.39	1.25–1.55	3.8 × 10⁻⁹
O2p (arm proximal)	0.99	0.79–1.24	0.94
O2x (arm mixed)	1.19	0.82–1.75	0.36
O3r (trunk/respiratory)	2.07	1.59–2.70	6.9 × 10⁻⁸
O4d (leg distal)	1.20	1.05–1.36	0.006
O4p (leg proximal)	1.22	0.96–1.55	0.104
O4x (leg mixed)	0.60	0.35–1.05	0.075
P1(n) (per +1 month)	0.967	0.963–0.970	< 10⁻⁶⁷
M_3class (ref = M0)			
M1d (UMN-predominant)	0.59	0.52–0.67	< 10⁻^15^
M2d (proximal/atypical LMN-predominant)	0.83	0.72–0.95	0.007
Demographics			
Age (per 10 years)	1.32	1.27–1.37	< 10⁻^4^⁶
Sex — male	1.07	0.99–1.16	0.089

Reference: O2d, M0, female sex, mean age

### Onset region (O)

After adjustment for P1(n), M_3class, age, and sex, three OPM_O categories carry an independent positive hazard relative to O2d (full HRs in Table 2, Supplementary Figure S2): O3r is the most aggressive onset region by a clear margin, followed by O1 and O4d. The remaining categories (O2p, O2x, O4p, O4x) are indistinguishable from O2d after adjustment, indicating that the prognostic signal previously associated with arm-proximal/scapulohumeral onset is no longer independently associated after adjustment for M_3class: the indolent course of flail-arm patients is captured by their classification as M2d rather than by their topography per se. The motor-neuron and onset effects are essentially unchanged when P is omitted from the model (O + M + age + sex; Table 3), confirming that the O–M structure does not depend on the propagation axis.

**Table 3:** Cox model with onset region (O) and motor-neuron pattern (M) but without the propagation axis (P), adjusted for age and sex (non-PLS cohort, *N* = 2738, events = 2561)

Variable	HR	95% CI	*p*
Onset region (ref. O2d arm-distal)			
O1 head/bulbar	1.36	1.22–1.52	< 0.001
O2p arm proximal	1.06	0.85–1.32	0.610
O2x arm mixed	1.17	0.80–1.71	0.418
O3r trunk/respiratory	2.10	1.61–2.74	< 0.001
O4d leg distal	1.07	0.94–1.21	0.328
O4p leg proximal	1.20	0.95–1.52	0.134
O4x leg mixed	0.65	0.37–1.13	0.128
Motor-neuron pattern (ref. M0 balanced)			
M1d UMN-predominant	0.58	0.51–0.66	< 0.001
M2d LMN-predominant	0.77	0.67–0.88	< 0.001
Covariates			
Age (per +10 years)	1.33	1.28–1.39	< 0.001
Sex (male)	1.11	1.02–1.20	0.014

The motor-neuron hazard ratios are essentially unchanged from the full OPM model (Table 2), showing that the prognostic effect of M is not an artefact of propagation timing. Reference categories: O2d (arm-distal), M0 (balanced UMN+LMN), female sex. Apparent C-index 0.639 without *P*, versus 0.706 for the full OPM model including P

### Propagation time (P)

P1(n) carries a strong negative hazard per +1 month (HR 0.967, 95% CI 0.963–0.970, *p* < 10⁻⁶⁸); for a 12 month difference in propagation time, the adjusted hazard ratio is approximately 0.66. P1(n) remains the dominant prognostic ingredient of the OPM model, as previously reported on PARALS.

### Motor-neuron axis (M)

Both motor-neuron classes are independently protective relative to M0 (M1d HR 0.59; M2d HR 0.83; full HRs in Table 2), and the effects are independent of OPM_O, P1(n), age, and sex. The contrast between M1d and M2d replicates the survival pattern observed in the unadjusted full-cohort analysis and supports the rationale for separating these two phenotypes from the M0 bulk.

### Internal validation

On the primary analytic dataset (*N* = 2738), bootstrap optimism-correction and repeated fivefold cross-validation confirmed minimal overfitting (optimism < 0.003 for both OPM5 and OPM6; full details in Supplementary Table S3, Supplementary Figure S3 and Supplementary Note S1). The optimism-corrected C-index was 0.703 for OPM5 and 0.705 for OPM6.

### Sensitivity analysis with M_4class (inclusion of the PLS-spectrum M1p subgroup)

To assess robustness to exclusion of the PLS-spectrum subgroup, we re-fitted OPM5 on the full non-cognitive cohort (*N* = 2802, events = 2590) including M1p as a fourth M class. As expected, M1p carried the strongest protective hazard (HR 0.16, 95% CI 0.11–0.23 versus M0, *p* < 10⁻^21^), consistent with the markedly longer survival of PLS-spectrum patients. Crucially, the HRs of M1d and M2d versus M0 were essentially unchanged (M1d 0.59; M2d 0.83), confirming that the principal finding—the prognostic separation of M2d from the M0 bulk—is robust to inclusion or exclusion of the PLS-spectrum subgroup. Full HRs are reported in Supplementary Table S4; the methodological reasons why M1p is unsuitable for a time-from-onset Cox model are discussed below.

### Comparison with alternative phenotypic taxonomies

A formal Cox model comparison against alternative phenotypic and diagnostic classifications on the same non-PLS cohort (*N* = 2738, events = 2561) is provided in Supplementary Table S5 and Supplementary Figure S4. OPM 3.3 substantially outperformed the classical bulbar/spinal binary categorisation (LR *χ*^2^ = 112.4, df = 8, *p* < 10⁻^2^⁰; ΔAIC = −96.4) and performed comparably to the classical phenotype classification (ΔAIC = −3.1, LR *χ*^2^ = 9.1, df = 3, *p* = 0.03), a difference negligible in effect size and indicating that the two phenotypic taxonomies convey largely overlapping prognostic information on this cohort. El Escorial diagnostic certainty remained complementary to the OPM phenotypic axes: combining OPM 3.3 with El Escorial yielded the highest discrimination of any model (C-corrected = 0.7128).

### Comparison with staging systems

The King’s clinical staging system [12] classifies ALS patients by the current extent of clinical involvement rather than by phenotype, and is therefore conceptually distinct from OPM. Using the King’s value at the visit closest to diagnosis (within ±6 months; coverage 99.8%; *N* = 2733, events = 2557) as an at-baseline staging covariate (Table 4), King’s-baseline alone reached a slightly higher bootstrap-corrected C-index than OPM 3.3 alone, and the combined model (OPM 3.3 + King’s-baseline) substantially outperformed either taxonomy alone (both nested likelihood-ratio tests highly significant; Table 4). Both M_3class hazard ratios remained significant after adjustment for King’s-baseline (M1d HR = 0.58; M2d HR = 0.85, *p* = 0.021), indicating that OPM phenotype and staging encode complementary prognostic information. Throughout these comparisons, P1(n) was held fixed across models to isolate whether O and M add prognostic information beyond disease burden at presentation.

**Table 4:** Cox model comparison of OPM 3.3 against clinical staging systems (King’s, MiToS, FT9)

Staging	*N*	Events	C (staging alone)	C (OPM alone)	C (combined)	Combined vs OPM 3.3	M1d HR (combined)	M2d HR (combined)
King’s	2733	2557	0.701	0.704	0.709	*χ*^2^ = 41.2, df = 3, *p* = 5.9 × 10⁻⁹, ΔAIC = −35.2	0.58 (0.51–0.66)	0.85 (0.74–0.98), *p* = 0.021
MiToS	2728	2552	0.703	0.705	0.713	*χ*^2^ = 59.5, df = 4, *p* = 3.8 × 10⁻^12^, ΔAIC = −51.5	0.60 (0.53–0.68)	0.86 (0.75–0.99), *p* = 0.033
FT9	2721	2549	0.702	0.704	0.711	*χ*^2^ = 73.9, df = 4, *p* = 3.4 × 10⁻^15^, ΔAIC = −65.9	0.59 (0.52–0.67)	0.86 (0.75–0.98), *p* = 0.026

For each staging system, nested Cox models were fitted on the non-PLS cohort with available staging: staging alone, OPM 3.3 alone, and the combined model, all anchored on P1(n), age and sex. Likelihood-ratio statistics and ΔAIC compare the combined model against OPM 3.3 alone

All models adjusted for P1(n) capped at 60 months, age (per 10 years) and sex. Reference categories: O2d (OPM_O), M0 (OPM_M_3class), female sex, and stage 1 for each staging system. C (combined) = optimism-corrected Harrell concordance (Harrell–Steyerberg bootstrap, *B* = 200). M1d and M2d hazard ratios are from the combined model and remain significant after adjustment for staging

Two parallel sensitivity analyses on the MiToS [13] and FT9 [14] staging systems were conducted on the same analytic cohort and reproduced the same qualitative pattern. Combining OPM 3.3 with either staging system significantly improves discrimination over OPM 3.3 alone (MiToS combined model: ΔAIC = −51.5; FT9 combined model: ΔAIC = −65.9), and the M_3class hazard ratios for M1d and M2d versus M0 remain stable across all three staging frameworks (M1d HR ranging 0.58–0.60; M2d HR ranging 0.85–0.86 across the King’s, MiToS and FT9 combined models). Full results are reported in Supplementary Tables S7 and S8.

## Discussion

In this large population-based incident cohort, the full three-axis ALS-OPM 3.3 specification [16, 17], with the motor-neuron axis operationalised as M_3class, achieved TRIPOD-compliant discrimination and separated three survival strata. OPM 3.3 did not materially increase prognostic discrimination over the classical phenotype classification (optimism-corrected C 0.7017 vs 0.7010), and did not surpass clinical staging or El Escorial-Awaji certainty. This apparent negative result proved to be the central finding of the study. We interpret it not as a limitation but as evidence that established phenotypes and OPM are not alternative ways of classifying ALS: rather, OPM resolves the prognostic signal already carried by classical phenotypes into more elementary, separately interpretable components—onset anatomy, propagation kinetics, and motor-neuron pattern—of which propagation is the principal contribution.

The three M classes have an immediate biological interpretation. M0 is the Charcot archetype of ALS, with simultaneous upper and lower motor-neuron involvement. M1d is the UMN-predominant variant, long recognised as the most indolent phenotype within the ALS spectrum [23, 24]; our adjusted HR of 0.59 versus M0 is consistent with this literature. M2d captures mostly, but not exclusively, the proximal/atypical LMN-predominant ‘flail-arm’ and ‘flail-leg’ variants, with a more indolent course than classical mixed ALS [9, 25, 26]; our adjusted HR of 0.83 versus M0 is consistent with this advantage. The M axis thus captures the three best-populated of the six ALS-OPM 3.3 M categories, mapping onto three well-established phenotypic strata already characterised in the ALS literature.

The PLS-spectrum subgroup (M1p) deserves some methodological considerations. Including M1p in a Cox model anchored on symptom onset introduces two interrelated biases, immortal-time bias and classification informed by outcome, because the ≥48 month qualifying period is event-free by construction and the class label depends on the observed trajectory; its HR of 0.16 (Supplementary Table S4) is therefore descriptive of the subgroup conditional on having reached M1p rather than a causal phenotype effect. Unlike the propagation axis, for which we apply a landmark approach (Supplementary Table S6), M1p remains outcome-dependent by definition, and a full treatment by competing-risks modelling is left to dedicated work. The lower motor-neuron counterpart, the PMA-spectrum M2p subgroup, raises a parallel and long-debated question, namely whether progressive muscular atrophy is a distinct entity or part of the ALS continuum; because a proportion of PMA patients develop upper motor-neuron signs or TDP-43 pathology consistent with ALS [29], we treated M2p as a pure-LMN pole of the same spectrum rather than as a separate disease, and analysed it descriptively alongside M1p.

The formal comparison against alternative classifications (Supplementary Table S5, Supplementary Figure S4) positions ALS-OPM 3.3 within the landscape of ALS taxonomies. OPM substantially outperforms the bulbar/spinal binary, replacing a 1-degree-of-freedom predictor with a structured stratification that recovers clinically meaningful sub-strata previously collapsed into the spinal pool. The classical phenotype classification achieves comparable overall discrimination by combining onset topography and motor-neuron pattern into single composite labels (such as ‘flail-arm’); the contribution of OPM is not a higher C-index but the disaggregation of that same prognostic signal into three clinically and prognostically separable dimensions, onset anatomy (O), propagation kinetics (P), and motor-neuron pattern (M), each independently interpretable. Unexpectedly, El Escorial revised diagnostic certainty attains a higher optimism-corrected C-index than any single phenotypic taxonomy, including OPM 3.3, because it largely reflects baseline multifocality, largely independent of OPM_O and M_3class. Its formal integration with the phenotypic axes warrants dedicated work. Because our cohort spans 2000–2022 and the graded El Escorial levels were recorded throughout, we used diagnostic certainty as originally graded; the Gold Coast criteria, which collapse these levels into a single diagnostic category, do not provide the certainty gradient that carries the complementary prognostic signal observed here, and their prognostic role is a question for prospectively Gold Coast-classified cohorts.

Crucially, the motor-neuron axis is not an artefact of propagation timing. With P removed, the M1d and M2d hazard ratios are essentially unchanged (Table 3), and they persist after adjustment for El Escorial certainty. El Escorial and OPM are therefore complementary rather than competing, El Escorial largely reflecting baseline multifocality and OPM the motor-neuron pattern, and the two combined exceed either alone (Supplementary Table S5). The higher discrimination of El Escorial reflects disease burden at presentation and the addition of neurophysiological information rather than a superior phenotypic taxonomy.

The decomposition reveals that the three axes contribute very unequally to prognosis. Onset anatomy alone discriminates only modestly (apparent C = 0.57); adding propagation kinetics raises discrimination substantially (to C = 0.68), whereas the motor-neuron pattern and demographics add comparatively little (to C = 0.71). Propagation is therefore the principal prognostic contribution of the OPM framework. Consistent with evidence that anatomical spread predicts survival, Tortelli et al. showed that time-to-generalisation predicts outcome [27], and Manera et al. quantified regional spread at diagnosis in PARALS itself [28]. OPM_P operationalises this by anchoring spread to a single, well-defined ALSFRS-R-based transition event. Crucially, this effect is not an artefact of survivorship: patients whose disease had already propagated to a second region carried a higher hazard at successive landmarks (HR 1.69 and 1.68; Supplementary Table S6), an analysis immune to immortal-time bias by construction. Although the O- and M-axes are defined as conceptually distinct, in population-based data, they show substantial biological coupling (O × M Cramér’s V = 0.51), whilst each retains independent prognostic information (likelihood-ratio *p* < 10⁻⁹ in both directions)—partially correlated, but not redundant.

Notably, the revised ALS-OPM classification itself recognises that motor-neuron involvement may evolve over the disease course, with possible M_3class reclassification at follow-up [17]. Our cross-sectional assignment captures the motor-neuron pattern at the first observation; the temporal dynamics of M, and the question of whether a shift towards LMN predominance acquires prognostic value, represent a natural longitudinal extension of the present work.

**Fig. 2:**
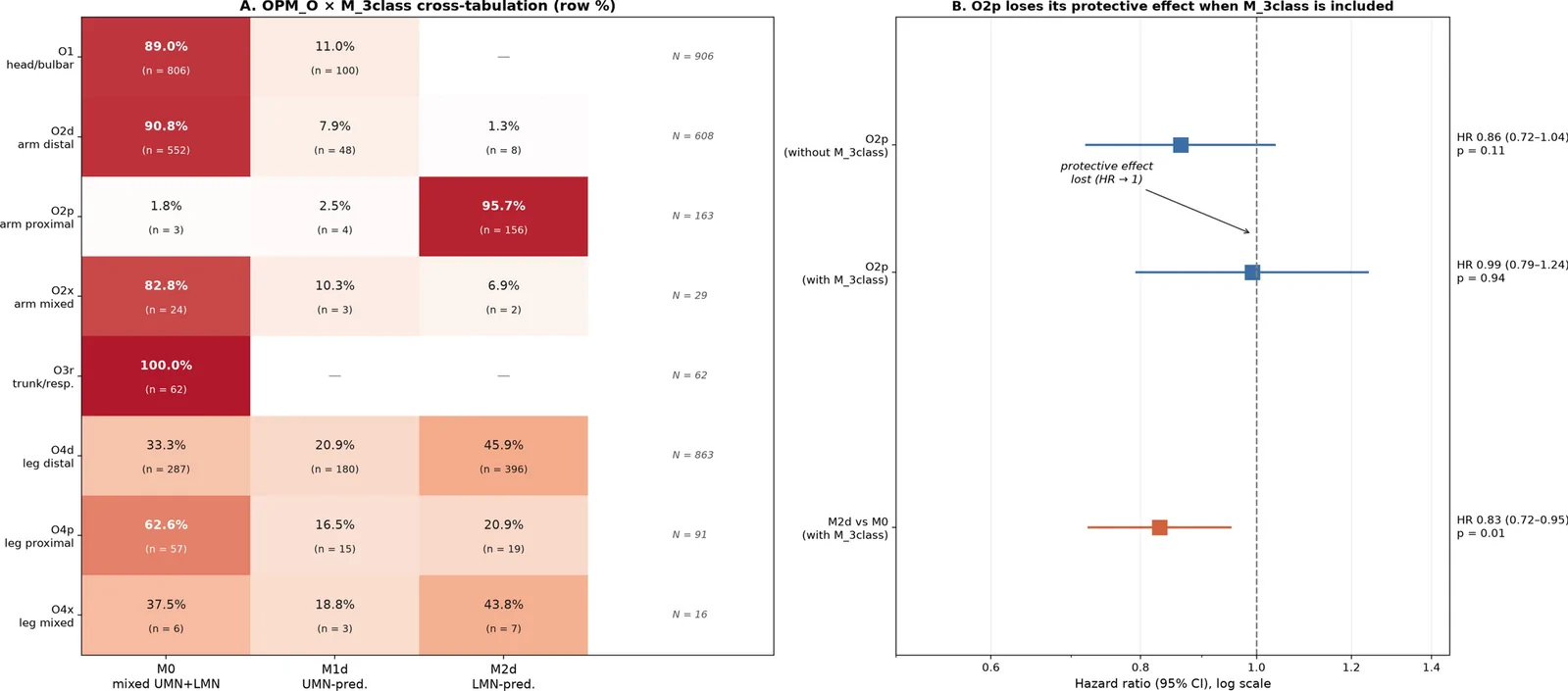
Cross-axis structure of ALS-OPM 3.3: anatomy (O) decodes through motor-neuron pattern (M). **a** OPM_O × M_3class cross-tabulation on the primary analytic cohort (N = 2738; M1p excluded). Cell colour intensity is proportional to the row percentage of M_3class within each OPM_O category. The cross-tabulation makes explicit several structural features: O3r (trunk-respiratory) is 100% M0, the O2p cell (arm-proximal/scapulohumeral) is 156/163 = 95.7% M2d (the ‘flail-arm’ cell, highlighted in red), and O4d (leg-distal) is the most heterogeneous category, splitting approximately 33%/21%/46% across M0/M1d/M2d. **b** Hazard ratios for O2p versus O2d (reference) and for M2d versus M0 in three Cox model specifications on the same cohort. Without inclusion of M_3class, the O2p category shows HR = 0.86 (95% CI 0.72–1.04, *p* = 0.11). After inclusion of M_3class, the O2p hazard ratio loses its protective effect and rises to 0.99 (95% CI 0.79–1.24, *p* = 0.94), losing all significance, whilst the M2d term against M0 retains HR 0.83 (95% CI 0.72–0.95, *p* = 0.01). The disappearance of the protective O2p effect when M_3class is included demonstrates that the indolent prognosis previously attributed to arm-proximal onset (an anatomical feature) is in fact a property of the underlying M2d motor-neuron pattern, which arm-proximal onset signals on a near-deterministic basis (95.7% of O2p patients are M2d)

The clearest single demonstration of this decomposition is that the prognostic effect of onset anatomy largely dissolves once motor-neuron pattern is introduced into the model. The arm-proximal cell illustrates this most clearly: arm-proximal onset behaves as a topographic surrogate for the LMN-predominant phenotype (Fig. 2), so its apparently favourable prognosis is a property of motor-neuron pattern rather than of onset anatomy. In clinical terms, a patient with arm-proximal onset should be counselled that the relatively favourable trajectory reflects the underlying LMN-predominant pattern, not the onset site itself, a caution that applies to any purely anatomical classification.

For the clinician, the practical value of OPM 3.3 is not that it predicts survival better than the classifications already in use, but that it makes explicit which component of the phenotype carries the prognostic information. Two patients of the same age and King’s stage may differ in motor-neuron pattern, and it is that difference, rather than the site of onset that first draws clinical attention, that separates their expected course. Recognising the motor-neuron axis as the operative one supports more accurate counselling at first presentation, and offers a principled basis for stratification in clinical trials, where balancing arms on motor-neuron pattern rather than on onset topography would reduce prognostic imbalance that the classical labels leave hidden.

**Fig. 3:**
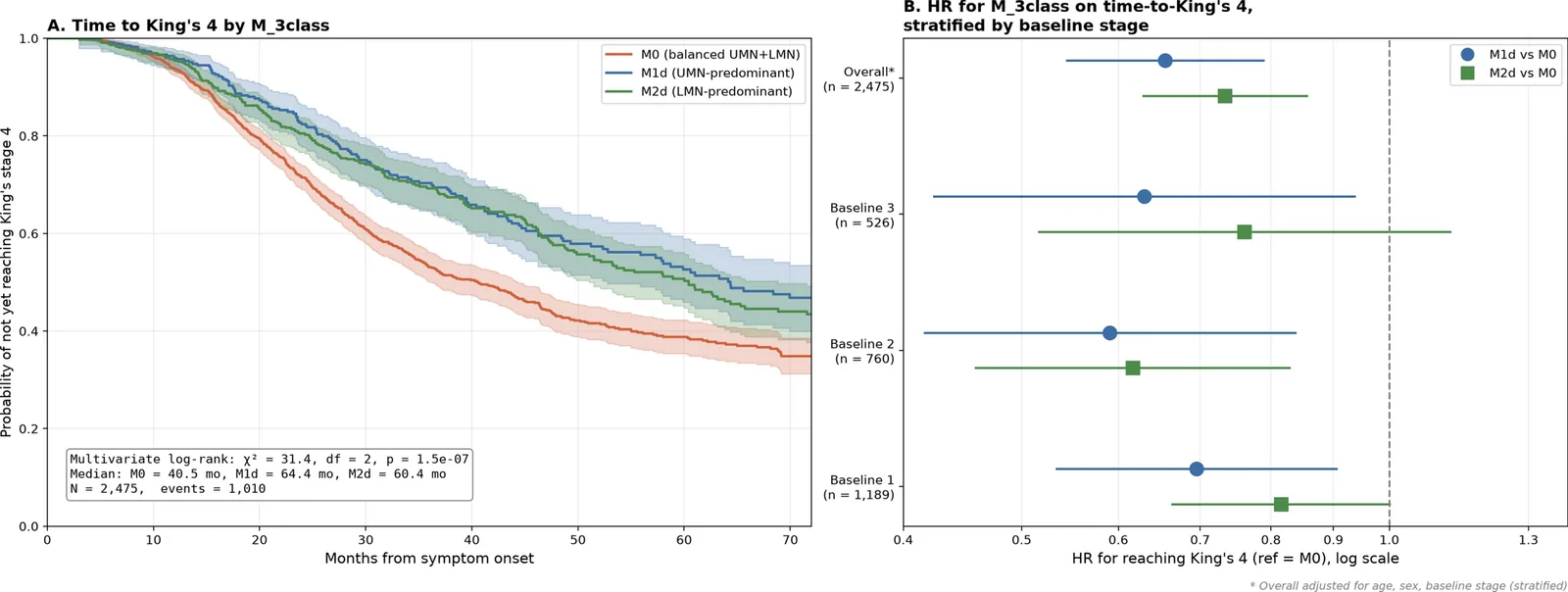
Time from symptom onset to King’s stage 4, stratified by M_3class. **a** Kaplan–Meier curves showing the probability of not having reached King’s stage 4, defined as the first recorded clinical visit with King’s = 4 (requirement of gastrostomy or non-invasive ventilation). Analysis restricted to patients at risk at diagnosis, i.e., King’s-baseline < 4 (*N* = 2475; 1010 events). Median time to King’s 4: M0 = 40.5 months (*n* = 1610, events 655); M1d = 64.4 months (*n* = 331, events 137); M2d = 60.4 months (n = 534, events 218). Multivariate log-rank *χ*^2^ = 31.4, df = 2, *p* = 1.5 × 10⁻⁷. Pairwise: M0 vs M1d and M0 vs M2d both *p* < 0.001; M1d vs M2d *p* = 0.32. **b** Forest plot of Cox hazard ratios for reaching King’s 4 by M_3class (reference = M0), separately within each King’s-baseline stratum and in the overall at-risk cohort adjusted for age, sex and King’s-baseline. Across strata King’s-baseline = 1, 2, 3, both M1d and M2d hazard ratios remain consistently < 1, demonstrating that OPM phenotype predicts the rate of clinical progression to King’s stage 4 independently of stage at presentation. The reference category for this analysis is M0 within each King’s-baseline stratum

OPM 3.3 is complementary to clinical staging across King’s, MiToS and FT9. The distinction is conceptual: staging measures how far the disease has spread, whereas OPM characterises which motor neurons are affected and in what topographic and kinetic pattern. Each staging system has practical limitations, MiToS being weighted towards advanced disease and King’s towards its earlier phases, with progression through stages not always sequential [14]; none captures the rate of progression or the motor-neuron phenotype at presentation, which is precisely the information the OPM axes add. Figure 3 provides a direct test: even at identical King’s baseline stages, M1d and M2d patients transition more slowly to severe milestones than M0, a pattern stable across MiToS and FT9 (Supplementary Tables S7 and S8).

Internal validation supports the model (Supplementary Note S1). The spline specification of propagation time (OPM6) yields only a marginal, non-significant gain over the linear specification (Table S2); on grounds of parsimony, we therefore recommend OPM5, with propagation capped at 60 months and modelled linearly, as the primary specification for clinical use and external validation.

This separation may be relevant to mechanistic studies and may facilitate biologically informed trial stratification: two patients matched on age, ALSFRS-R, and King’s stage but differing on M may represent distinct disease patterns that traditional phenotypic labels would conflate.

Limitations include the population-based but single-region nature of the cohort, which calls for external validation of M_3class in other incident ALS registries. In the PARALS dataset, coverage of P1(n) on the analytic dataset reached 100.0%. Finally, the M_3class operationalisation relies on the availability of a registry-level classification of upper and lower motor-neuron involvement; its applicability to other registries depends on equivalent characterisation of the motor-neuron phenotype (PLS, PMA, UMN-predominant, LMN-predominant, balanced). Because P reflects post-onset disease spread by construction, the propagation axis incorporates information accruing after onset; however, its timing was dated primarily from the patient’s history per the ALS-OPM 3.3 specification, and the landmark analysis (Supplementary Table S6) confirmed that the principal findings are robust to the treatment of the propagation axis [28]. The reliance on patient-reported timing for part of the propagation data is a limitation intrinsic to any onset-anchored study, and we addressed it by dating propagation primarily from the clinical history but confirming it prospectively where follow-up was available; the concordance between the two sources, and the robustness of the landmark analysis, make it unlikely that recall imprecision materially altered the propagation estimates.

In this population-based incident cohort, OPM 3.3 did not materially improve prognostic discrimination over established phenotypic classifications, clinical staging, or El Escorial-Awaji certainty, with propagation emerging as the dominant prognostic dimension. The prognostic information carried by classical phenotypes is not lost within OPM but redistributed across anatomical, temporal, and motor-neuron dimensions. Although observational data cannot establish mechanistic causality, the present analyses support the interpretation that OPM provides a framework for understanding what prognostic information classical phenotypes actually encode. Like the staging systems it is measured against, OPM 3.3 is a purely motor classification, and does not capture the extra-motor dimensions of the disease, cognitive and behavioural involvement across the ALS-frontotemporal spectrum, and less commonly extrapyramidal or cerebellar features, which are clinically relevant to prognosis and to the burden on patients and caregivers; whether these domains represent complementary sources of phenotypic heterogeneity that a future extension of OPM should incorporate remains an open question.

## Supplementary Information

Below is the link to the electronic supplementary material.

Supplementary file1 (DOCX 808 KB)

## Data Availability

The PARALS clinical data and the code reproducing the analyses are available from the corresponding author upon reasonable request, subject to ethical approval.
