# Supplementary material for: What do ALS motor phenotypes measure? A population-based decomposition of their prognostic dimensions
Source: J Neurol. 2026 Sep 18;273(10):604. doi: 10.1007/s00415-026-14134-z (PMC13588936; doi:10.1007/s00415-026-14134-z)
Supplement: Supplementary file 1 [file 415_2026_14134_MOESM1_ESM.docx]

**Supplementary Methods**

**ALS-OPM 3.3 classification — extended definitions**

*Axis structure.* The three ALS-OPM 3.3 axes are defined by Meyer et al. as conceptually distinct determinants. In the present population-based cohort the axes proved partially correlated while retaining complementary prognostic information: strong correlation between two axes does not preclude their being biologically and prognostically distinct (the O–M association is quantified in the main text, Cramér’s V = 0.51).

*Onset region (O).* Taken from the region of first motor involvement recorded in the registry, yielding eight categories: O1 head/bulbar; O2d arm, distal; O2p arm, proximal/scapulohumeral; O2x arm, mixed; O3r trunk/respiratory; O4d leg, distal; O4p leg, proximal; O4x leg, mixed. Full clinical definitions are given in the table below.

*Propagation (P1(n)).* The interval in months from symptom onset to involvement of a second, vertically distant body region, capped at 60 months for the Cox specification. Following the ALS-OPM 3.3 specification, its temporal qualifier was dated primarily from the patient’s history (the anamnestic date of second-region involvement); for patients in whom propagation had not yet occurred at the first visit, it was dated prospectively from the first follow-up ALSFRS-R assessment at which a non-onset region subscore fell below 4. Patients with established propagation but no datable timing were classified P1(x). The anamnestic and prospective timings showed strong rank concordance (Spearman ρ = 0.785; Supplementary Table S1). The four reference regions are bulbar, cervical (upper limb), thoracic (trunk/respiratory) and lumbosacral (lower limb). O and M are baseline descriptors, whereas P incorporates the post-onset kinetics of regional spread.

*Motor-neuron pattern (M).* Taken from the registry’s existing classification of upper and lower motor-neuron involvement and operationalized as a three-level variable M_3class (M0, balanced UMN+LMN; M1d, UMN-predominant; M2d, LMN-predominant), covering 99% of the non-PLS analytic cohort. The rare pure-LMN (M2p) and dissociated UMN/LMN (M3) configurations were not retained as separate classes for multivariable modelling due to insufficient numerosity and diagnostic uncertainty. As such, M2p (including only 17 cases) was collapsed in M2d; M3 (9 cases) was collapsed in M0. M1p (PLS-spectrum, n = 64) was analyzed separately to avoid immortal-time bias arising from the long survival required to fulfil PLS diagnostic criteria. Full clinical definitions are given in the table below.

*Onset axis (OPM_O), 8 categories.*

| **OPM_O** | **Topographic label** | **Clinical definition (region of first motor involvement)** |
| --- | --- | --- |
| O1 | Head / bulbar | Onset in the bulbar region (dysarthria and/or dysphagia) |
| O2d | Arm — distal classic | Onset in the upper limb with a distal predominance (hand/forearm) |
| O2p | Arm — proximal | Onset in the upper limb with a proximal/scapulohumeral predominance |
| O2x | Arm — mixed / non-classifiable | Onset in the upper limb without a documented distal/proximal predominance |
| O3r | Trunk / respiratory | Onset in the trunk or with respiratory/axial involvement |
| O4d | Leg — distal pattern | Onset in the lower limb with a distal predominance (foot/leg) |
| O4p | Leg — proximal pattern | Onset in the lower limb with a proximal predominance (thigh/pelvic girdle) |
| O4x | Leg — mixed / non-classifiable | Onset in the lower limb without a documented distal/proximal predominance |

*Motor-neuron pattern axis (M_3class).*

| **M_3class** | **Label** | **Clinical definition** |
| --- | --- | --- |
| M0 | Balanced UMN + LMN (classic mixed) | Simultaneous upper and lower motor-neuron signs (classic ALS) |
| M1d | Dominant UMN (UMN-predominant) | Predominant upper motor-neuron signs, not meeting PLS criteria |
| M2d | Dominant LMN, proximal/atypical | Predominant lower motor-neuron signs with proximal/atypical distribution (scapulohumeral / flail-arm and flail-leg variants) |
| M2p | Pure LMN (PMA-spectrum) | Pure lower motor-neuron involvement (progressive muscular atrophy; not retained for multivariable modelling) |
| M1p | Pure UMN (PLS-spectrum) | Pure upper motor-neuron involvement meeting PLS criteria (analysed separately; Table S4) |

**Supplementary Table S1. Validity of the propagation axis P1(n): concordance between the anamnestically dated propagation (primary, per the ALS-OPM 3.3 specification) and the prospective ALSFRS-R-based timing.** Among the 822 patients with both a dated anamnestic second-region involvement and a prospective ALSFRS-R-based estimate, the two measures showed strong rank concordance (Spearman ρ = 0.785), with the prospective estimate later on average (Bland-Altman bias +5.3 months, SD 6.7). Landmark analyses of propagation as a prognostic marker are reported separately in Supplementary Table S6.

| **Analysis** | **N** | **Statistic** | **Value** |
| --- | --- | --- | --- |
| Concordance: anamnestic vs prospective P1(n) | 822 | Spearman ρ | 0.785 |
|  |  | Bland-Altman bias (prosp − anamn) | +5.3 mo (SD 6.7) |

**Supplementary Table S2. Hierarchical Cox models on the primary analytic dataset (M_3class, M1p excluded; N = 2,738, events = 2,561).** k = number of free parameters; AIC penalises by 2k. The primary specification OPM5 is the principal Cox model reported in Table 2.

| **Model** | **Specification** | **logL** | **AIC** | **k** | **C-index** |
| --- | --- | --- | --- | --- | --- |
| OPM1 | OPM_O only | −17,987 | 35,988 | 7 | 0.5682 |
| OPM2 | + P1(n) linear | −17,783 | 35,583 | 8 | 0.6782 |
| OPM4 | + P + M_3class | −17,741 | 35,501 | 10 | 0.6853 |
| OPM5 | OPM_O + P + M_3class + age + sex | −17,630 | 35,285 | 12 | 0.7056 |
| OPM6 | OPM5 with P spline (df=4) | −17,628 | 35,285 | 14 | 0.7083 |

**Supplementary Table S3. Internal validation of the OPM Cox prognostic model (TRIPOD-compliant). N = 2,738; events = 2,561 (M1p excluded).** Apparent C = full-sample Cox concordance. Optimism = mean of (apparent C − test C) across 200 bootstrap resamples. CV mean = mean of 100 fold-level C-indices from 20 repetitions of 5-fold partitioning.

| **Model** | **Apparent C** | **Bootstrap optimism (B=200)** | **Optimism-corrected C** |
| --- | --- | --- | --- |
| Age + sex (baseline) | 0.5967 | −0.0006 | 0.5973 |
| OPM5 | 0.7056 | +0.0024 | 0.7032 |
| OPM6 (P spline) | 0.7083 | +0.0026 | 0.7057 |

5-fold CV mean (95% CI): baseline 0.596 (0.571–0.617); OPM5 0.702 (0.685–0.719); OPM6 0.704 (0.686–0.721).

**Supplementary Table S4. Hazard ratios in the sensitivity Cox model with M_4class (M1p) included; N = 2,802, events = 2,590.** Reference categories: O2d (onset), M0 (motor-neuron axis), female sex. Model: OPM5 with M_4class instead of M_3class. Apparent C = 0.7029; optimism-corrected C = 0.7011. M1p (PLS-spectrum) carries the largest protective HR (0.16) but its inclusion in a from-onset Cox model is methodologically problematic (see Discussion).

| **Variable** | **HR** | **95% CI** | **p-value** |
| --- | --- | --- | --- |
| O1 (head/bulbar) | 1.39 | 1.24–1.55 | <10⁻⁸ |
| O2p (arm proximal) | 1.00 | 0.80–1.25 | 0.99 |
| O2x (arm mixed) | 1.19 | 0.81–1.74 | 0.37 |
| O3r (trunk-respiratory) | 2.07 | 1.59–2.69 | <10⁻⁷ |
| O4d (leg distal) | 1.22 | 1.07–1.38 | 0.002 |
| O4p (leg proximal) | 1.20 | 0.95–1.53 | 0.128 |
| O4x (leg mixed) | 0.66 | 0.39–1.11 | 0.119 |
| P1(n) per +1 month | 0.967 | 0.963–0.970 | <10⁻⁷² |
| M1d (dominant UMN) | 0.58 | 0.51–0.66 | <10⁻¹⁶ |
| M1p (pure UMN, PLS) | 0.16 | 0.11–0.23 | <10⁻²¹ |
| M2d (proximal/atypical LMN) | 0.81 | 0.71–0.93 | 0.003 |
| Age (per 10 years) | 1.33 | 1.28–1.38 | <10⁻⁴⁷ |
| Sex (male) | 1.07 | 0.99–1.16 | 0.076 |

**Supplementary Table S5. Cox model comparison across alternative phenotypic taxonomies on the primary non-PLS ALS cohort (N = 2,728, events = 2,552; 10 patients excluded from the N = 2,738 analytic cohort for missing classic-phenotype or El Escorial classification at baseline).** C-corrected = bootstrap optimism-corrected Harrell C-index, estimated with B = 120 resamples uniformly across all competing models to ensure a like-for-like comparison; the OPM5 value reported here (0.7017) therefore differs marginally from the primary internal-validation estimate (0.703, B = 200; Supplementary Table S3), the small difference reflecting bootstrap sampling variation. All models adjusted for P1(n), age, sex. Model E* (primary) = OPM5. Model I combines B and E.

| **Model** | **Taxonomy** | **k** | **C-corr (95% CI)** |
| --- | --- | --- | --- |
| A | Bulbar / Spinal binary | 4 | 0.6953 (0.690–0.701) |
| B | El Escorial (DEF/PRO/PRL/POS/SOS) | 7 | 0.7059 (0.701–0.711) |
| C | Classical phenotypes (7-cat) | 9 | 0.7010 (0.696–0.706) |
| E* | OPM 3.3 (OPM_O 8-cat + M_3class) — primary | 12 | 0.7017 (0.697–0.707) |
| I | OPM 3.3 + El Escorial (combined) | 16 | 0.7128 (0.708–0.718) |

**Supplementary Table S6. Landmark analysis addressing immortal-time bias on the propagation axis.** At fixed landmarks from symptom onset (6 and 12 months), patients alive and under observation were classified by propagation status (already propagated versus not yet, dated anamnestically per the ALS-OPM 3.3 specification), and residual survival was compared between groups. This design is immune to immortal-time bias by construction. Patients already propagated at each landmark carried a higher subsequent hazard (HR ≈ 1.69 at 6 months, 1.68 at 12 months; both p < 10⁻³¹), confirming that the prognostic effect of propagation persists after removal of survivorship effects.

| **Landmark** | **N alive (propagated / not)** | **HR propagated vs not (95% CI)** | **p** | **Median survival (prop. vs not)** |
| --- | --- | --- | --- | --- |
| 6 months | 2,686 (884 / 1,802) | 1.69 (1.55–1.84) | 5.2×10⁻³⁴ | 22.0 vs 35.0 mo |
| 12 months | 2,383 (1,378 / 1,005) | 1.68 (1.54–1.83) | 2.7×10⁻³² | 27.9 vs 43.1 mo |

**Supplementary Table S7. Cox model comparison: OPM 3.3 versus MiToS-baseline staging (non-PLS ALS, N = 2,728, events = 2,552; reduced from N = 2,738 by absence of a MiToS-baseline within ±6 months of diagnosis).** Adjusted for P1 (capped 60 mo), age, sex. Mi2* = primary OPM 3.3. LR: Mi1+Mi2 vs Mi1 χ² = 118.0, df = 9, p = 3.4×10⁻²¹, ΔAIC = −100.0; vs Mi2* χ² = 105.1, df = 4, p = 7.9×10⁻²², ΔAIC = −97.1.

| **Model** | **Specification** | **k** | **logL** | **AIC** | **C-corr (95% CI)** |
| --- | --- | --- | --- | --- | --- |
| Mi1 | MiToS-baseline (4-cat, collapsed) | 6 | −16,871 | 33,754 | 0.700 (0.695–0.704) |
| Mi2* | OPM 3.3 (primary) | 11 | −16,871 | 33,763 | 0.702 (0.697–0.707) |
| Mi1+Mi2 | OPM 3.3 + MiToS-baseline (combined) | 14 | −16,813 | 33,654 | 0.706 (0.703–0.710) |

**Supplementary Table S8. Cox model comparison: OPM 3.3 versus FT9-baseline staging (Fine’til 9; Thakore et al., 2018). Non-PLS ALS cohort N = 2,721, events = 2,549.** Adjusted for P1 (capped 60 mo), age, sex. F2* = primary OPM 3.3. LR: F1+F2 vs F1 χ² = 125.5, df = 9, p = 8.8×10⁻²², ΔAIC = −107.5; vs F2* χ² = 191.3, df = 4, p = 8.2×10⁻⁴¹, ΔAIC = −183.3 (largest of the three staging systems).

| **Model** | **Specification** | **k** | **logL** | **AIC** | **C-corr (95% CI)** |
| --- | --- | --- | --- | --- | --- |
| F1 | FT9-baseline (5-cat, 0–4) | 7 | −16,833 | 33,679 | 0.706 (0.702–0.709) |
| F2* | OPM 3.3 (primary) | 11 | −16,871 | 33,763 | 0.702 (0.697–0.707) |
| F1+F2 | OPM 3.3 + FT9-baseline (combined) | 15 | −16,774 | 33,577 | 0.712 (0.708–0.715) |

**Supplementary Figure S1. Patient flow and selection of the primary analytic cohort.** Of 2,829 incident PARALS patients (2000–2022), 2,808 had an assignable OPM_O onset region and 2,802 had complete survival outcome; after excluding 64 M1p (PLS-spectrum) cases, the primary non-PLS analytic cohort comprised 2,738 patients (2,561 events). Secondary staging subsets: King’s 2,733; MiToS 2,728; FT9 2,721.

**Supplementary Figure S2.** Forest plot of hazard ratios in the OPM primary Cox model OPM5 (N = 2,738; events = 2,561; M1p excluded), corresponding to the estimates reported in Table 2 of the main text. Reference categories: O2d for OPM_O (arm distal), M0 for M_3class (balanced UMN+LMN), female sex, mean age. Adjusted hazard ratios are shown with 95% confidence intervals on a log scale; P1(n) (continuous, capped at 60 months) is shown as the hazard ratio per +1 month of propagation time.

**Supplementary Figure S3.** Forest plot of optimism-corrected Harrell C-index for alternative phenotypic taxonomies (N = 2,728, events = 2,552; 10 patients excluded for missing classic-phenotype or El Escorial classification). Models ordered by descending C-corrected. Error bars are 95% bootstrap CIs (B = 200). Reference dotted line at the primary model E (OPM 3.3, 0.702). The combined model (I) achieves the highest discrimination; El Escorial alone (B) outperforms every phenotypic-only model. All models adjusted for P1(n), age, sex.

**Supplementary Figure S4.** Time from symptom onset to MiToS stage 3, stratified by M_3class. a Kaplan–Meier curves for the probability of not yet reaching MiToS stage 3, restricted to patients at risk (N = 2,608; 517 events); median time-to-MiToS-3 was not reached in any group within follow-up. Multivariate log-rank χ² = 24.1, df = 2, p = 5.8×10⁻⁶. b Forest plot of hazard ratios for reaching MiToS stage 3 by M_3class (reference M0), overall (adjusted for age, sex and baseline stage) and within each MiToS-baseline stratum; both M1d and M2d show consistently lower hazards than M0.

**Supplementary Figure S5.** Time from symptom onset to FT9 stage 4, stratified by M_3class. a Kaplan–Meier curves for the probability of not yet reaching FT9 stage 4, restricted to patients at risk (N = 2,537; 888 events); median time-to-FT9-4: M0 = 51.0 months, M1d = 79.8 months, M2d = 73.6 months. Multivariate log-rank χ² = 19.9, df = 2, p = 4.7×10⁻⁵. b Forest plot of hazard ratios for reaching FT9 stage 4 by M_3class (reference M0), overall (adjusted for age, sex and baseline stage) and within each FT9-baseline stratum; both M1d and M2d show consistently lower hazards than M0.
